# SPHN Connector - a scalable pipeline for generating validated knowledge graphs from federated and semantically enriched health data

**DOI:** 10.1186/s12911-026-03383-7

**Published:** 2026-02-13

**Authors:** Vasundra Touré, Deepak Unni, Philip Krauss, Andrea Brites Marto, Katie Kalt, Nicola Stoira, Maximilian Pickl, Sabine Österle

**Affiliations:** 1 Swiss Personalized Health Network, SIB Swiss Institute of Bioinformatics, Basel, Switzerland; 2 Accenture AG, Zurich, Switzerland; 3 University Hospital Zurich, Zurich, Switzerland; 4 Accenture GmbH, Munich, Germany

**Keywords:** Knowledge graphs, Semantic web, Linked data, Data provisioning, Clinical real-world data

## Abstract

**Background:**

The integration and reuse of heterogeneous health data, including clinical records, cohort studies, and omics datasets, are essential for advancing modern biomedical research. Knowledge graphs offer a powerful means to semantically link such data, enabling interoperability and reuse. The Swiss Personalized Health Network has developed a comprehensive semantic interoperability framework to implement the FAIR (Findable, Accessible, Interoperable, Reusable) principles at a national level.

**Methods:**

This paper presents the strategy adopted and resulting SPHN Connector tool for enabling data providers to transform their local data into semantically enriched knowledge graphs following the RDF and related Semantic Web standards. Rather than requiring centralized data transformation, the SPHN Connector allows each institution to build knowledge graphs locally from their heterogeneous data sources, maintaining data governance at the source while ensuring semantic interoperability across sites.

**Results:**

The SPHN Connector tackles the technical challenges in federated knowledge graph construction. It converts diverse data formats into SPHN-compliant semantically enriched RDF, and offers capabilities for data transformation, de-identification, and validation, particularly for iterative deliveries.

**Conclusion:**

These generated datasets can then either be integrated centrally or used in a federated way, allowing for the linkage of information from the same patient, for example, clinical routine data and omics metadata, as well as the combination of data from different patients across sites.

## Background

Integrating and reusing heterogeneous health data, ranging from clinical routine records to cohort studies and omics datasets, is central to modern biomedical research [1]. This challenge is amplified in federated health data networks, where data remains under the control of local institutions due to legal, ethical, and governance constraints. Semantic knowledge graphs (KGs) have emerged as a powerful paradigm to link data, harmonizing data structures while preserving meaning through semantic representations. By representing data using explicit semantics with shared ontologies and encoding them in the Resource Description Framework (RDF) [2], KGs enable the harmonization of heterogeneous data sources while preserving contextual meaning and supporting reasoning-based queries. Several initiatives build biological and biomedical KGs using an Extract-Transform-Load (ETL) approach where data from heterogeneous sources are extracted, semantically enriched, and harmonized using domain-specific ontologies, and loaded into a graph-based store for querying and analysis. For instance, Bio2RDF [3] converts resources like HUGO Gene Nomenclature Committee (HGNC) [4] and DrugBank [5] into RDF, mapping identifiers to Uniform Resource Identifiers (URIs) and linking datasets for querying. The Monarch Initiative [6], which focuses on integrating cross-species genotype-phenotype data, harmonizes data using the Open Biological and Biomedical Ontologies (OBO) Foundry ontologies for phenotype-driven rare disease diagnosis and translational research [7, 8]. The Knowledge Graph Hub [9] offers reusable ETL components, where data sources are mapped to the Biolink Model [10], for an enriched KG with biomedical associations. Another example is PheKnowLator [11], which automates the construction of semantically rich KGs using ontologies and OWL reasoning. The Blue Brain Nexus [12] offers a scalable, provenance-aware linked data platform designed for neuroscience but also supports the broader biomedical domain. However, these approaches assume centralized data access, making them less suitable for federated environments.

An alternative approach relies on virtual KGs and Ontology-Based Data Access (OBDA), which enable on-demand querying of source systems without fully transforming data into RDF [13, 14]. Although effective in centrally managed environments, such approaches are incompatible within a federated context due to operational, infrastructural, and governance-related constraints. Data providers must materialize RDF triples locally before sharing them as files through a secure infrastructure, making virtualized access infeasible. Hospital systems are also heterogeneous with distinct internal infrastructures, data models, and clinical data platforms, making the creation and maintenance of individualized OBDA mappings impractical.

Despite the diversity and maturity of tools available for biomedical KG construction or federated querying, very few are designed to address data integration challenges of routine clinical data highlighted above. Most biomedical KGs are generated centrally, aggregating source data into a single repository before, during, or after transformation.

As an alternative to the approaches described above, common data models have been developed to support federated clinical analytics. The Observational Health Data Sciences and Informatics (OHDSI) network harmonizes distributed clinical databases into the Observational Medical Outcomes Partnership (OMOP) Common Data Model [15, 16] and enables federated analytics; while the European Health Data & Evidence Network (EHDEN) operates a large-scale OMOP-based privacy-preserving network for real-world evidence data. However, these models fall short in capturing the full semantic depth and flexibility required for advanced clinical data analytics. Specifically, OMOP-based pipelines lack native support for ontology-driven reasoning, dynamic extensibility for evolving clinical concepts, and seamless cross-domain linkage for richer semantic integration. Several research projects have demonstrated the value of transforming OMOP into KGs, unlocking capabilities that are difficult to achieve with OMOP alone [17–19].

In contrast, RDF-based KGs excel in these areas by design: their use of standardized Internationalized Resource Identifiers (IRIs) to uniquely identify resources and their relationships, enables seamless “plug and play” interoperability, allowing integration across diverse datasets and systems. For example, by integrating terminologies such as Systematized Nomenclature of Medicine - Clinical Terms (SNOMED CT [20]), hierarchical reasoning becomes possible, enabling the retrieval of all patients with diagnoses under a broad disease category (e.g., all subtypes of diabetes mellitus), or the identification of patients with disorders caused by a specific virus, leveraging the transversal relationships. These broad and transversal relationships (i.e., hierarchical classifications, causal links, conceptual associations) are not inherently captured in raw clinical data. Instead, they are defined in specialized terminologies, which must be interlinked with the data to enable efficient machine processing and unlock the full potential of the data.

Within the Swiss Personalized Health Network (SPHN), a comprehensive semantic interoperability framework has been developed to support the FAIR (Findable, Accessible, Interoperable, Reusable) principles [21]. This framework is centered around the SPHN RDF Schema [22], defining a Semantic Web-compliant blueprint for health data representation. Beyond the core schema, project-specific extensions enrich the SPHN RDF Schema with additional field-specific semantics. Both core and extended schemas are complemented by Semantic Web artifacts such as Shapes Constraint Language (SHACL [23]) validation rules and SPARQL Protocol and RDF Query Language (SPARQL [24]) data exploration queries, automatically generated with the SPHN Schema Forge tool [25]. Standard terminologies, such as SNOMED CT [20], Logical Observation Identifiers Names and Codes (LOINC) [26], Anatomical Therapeutic Chemical (ATC) [27], and International Statistical Classification of Diseases and Related Health Problems 10th revision German modification (ICD-10-GM) [28], are provided in RDF with the DCC Terminology Service [29]. Using these resources, data providers transform local datasets into RDF representations that conform to the SPHN RDF Schema for interoperability and reusability.

The SPHN RDF Schema defines interoperable data semantics but does not guide hospitals on transforming or validating local datasets (e.g., conventions for IRI generation). Each data provider relies on its own clinical data platform and originally had to maintain separate local pipelines for the RDF generation. The data comes in diverse formats (CSV, relational databases, JSON [30], and, in the future, also FHIR [31]). Combined with the need for technical validation to enhance data quality and apply de-identification measures such as patient pseudonymization and date shifting at scale [32], these pipelines become complex. The variability observed in outputs made it challenging to integrate data into a single knowledge graph. Limited expertise in KG technologies among data providers further complicated these implementations. Consequently, stakeholders have expressed the need for structured guidance and practical tools.

To address this, SPHN developed the SPHN Connector, a unified tool that produces consistent, semantically structured, validated, and de-identified data aligned with the SPHN Semantic Interoperability Framework. While the SPHN Connector automates RDF generation, validation, and pseudonymization, the upstream mapping of local codes to standard terminologies (e.g., SNOMED CT, LOINC) remains a time-consuming, manual and expert-driven process. This distinction is critical: the SPHN Connector lowers technical barriers to data harmonization after semantic mapping has been completed. By automating key steps, the SPHN Connector lowers technical barriers to semantic data processing and enables scalable production of FAIR, high-quality data for federated KG construction. Although currently implemented within the SPHN network, the architecture of the SPHN Connector demonstrates general principles for federated, schema-driven RDF generation. These principles are adaptable to other national or institutional settings, enabling broader applications.

**Fig. 1:**
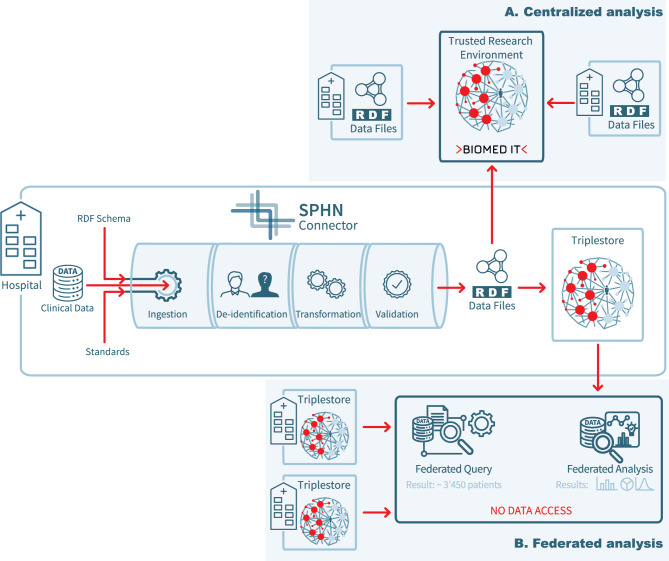
Centralized versus federated approach for the use of knowledge graphs generated by the SPHN Connector. (**A**) Centralized analysis of the RDF files generated by data providers. Data is transferred to a trusted research environment (e.g. BioMedIT [33]) where users can safely perform their research studies. (**B**) Federated analysis of knowledge graphs built at data providing institutions. These graphs are stored in a triple store locally, and aggregated results can be accessed in a federated manner, allowing users to assess the feasibility of research projects based on the data available at each institution

The SPHN Connector supports two complementary use case scenarios highlighted in Fig. 1. Datasets can be securely transmitted to trusted research environments, such as BioMedIT [33], for centralized processing and integration (Fig. 1A). Alternatively, in the federated analysis scenario (Fig. 1B), the KG is conceptually unified according to the SPHN RDF Schema but stored locally at participating hospitals, allowing data to remain on-site while enabling cross-institutional analyses. In both scenarios, the SPHN Connector provides syntactic and semantic foundations for conceptual unification, achieved through (i) the shared SPHN RDF Schema and (ii) a consistent, globally unique IRI naming and identifier management strategy. These guarantees enable either centralized merging or federated querying, depending on the requirements of the research project.

In this paper, we present the SPHN Connector, describe its design and workflow; and discuss how it facilitates the KG construction across Swiss hospitals for generating a consistent and semantically valid RDF data.

## Methods

The local generation of semantically rich KGs aligned here with the SPHN Semantic Interoperability Framework was designed to address key challenges and stakeholder requirements for the downstream integration of heterogeneous health data across institutions.

We identified three generally applicable dimensions of challenges associated with distributed KG creation:

- **Data heterogeneity:** Distributed KG generation requires integrating data from diverse sources that often differ in format and structure. Additional challenges come from differences in language, semantic representation, availability of contextual information, and overall data quality.
- **Heterogeneous infrastructures and tooling:** In distributed environments, data and processing pipelines are often heterogeneous, even when designed to generate data conforming to a specific model. Data extraction and transformation tools rely on their own internal data models, which can affect how source data is mapped and transformed.
- **Variability in user background:** Stakeholders involved in KG generation are often data engineers, data scientists, and software developers. Their approach to building local data processing pipelines varies depending on technical expertise, understanding of the underlying semantics, and familiarity with the target data model. This highlights the need for a robust, automated, reproducible, and well-documented workflow.

These challenges were identified through stakeholder discussions and requirements gathering. Guided by these insights, we describe below the resulting conceptual strategies and their corresponding technical implementations in the SPHN Connector.

### General tool requirements

#### Data ingestion and automated transformation

SPHN requires that project data is provided in RDF format and delivered to researchers as materialized RDF files. Data providers therefore requested a tool that partially automates the transformation of system-aligned input data formats into RDF, removing the need to develop and maintain custom RDF transformation pipelines. To this end, the SPHN Connector provides dedicated ingestion interfaces for JSON, tabular formats (CSV/Excel), and relational database management systems (RDBMS). Importantly, data providers remain responsible for the upstream semantic mapping of their local data, including mapping local codes and structures to standard terminologies (e.g. SNOMED CT, LOINC) and to the SPHN Concepts. Once this expert-driven mapping is completed and data is aligned with the requirements of ingestion templates, the SPHN Connector performs the automated downstream transformation and validation of the data into RDF.

**Fig. 2:**
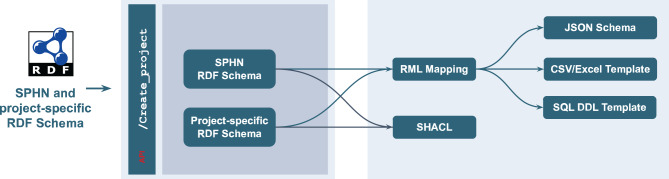
Configuration step with Create_project API endpoint. From the RDF schema (SPHN or project-specific) provided by the user, the SPHN Connector generates the input template interfaces for each of the following supported formats: JSON, CSV/Excel, RDBMS (with an SQL data Definition Language (DDL) template) using an RML mapping. Additionally, SHACL rules are derived using the SPHN SHACLer

The schema serves as the sole source of truth for all data-related components within the SPHN Connector. From it, key components such as ingestion templates, transformation logics, and validation rules are automatically derived (see Fig. 2).

#### Multi-project handling

The SPHN Connector is designed to support multiple projects within the same installation at each site, accommodating different schema versions and project-specific schemas. Projects remain strictly isolated from one another in the tool with respect to their schema definitions, patient identifiers, database views, and populated tables. For example, when a patient must be provided to two different projects, this patient will be processed separately within each project. However, within a project, configuration and de-identification rules persist across data deliveries, ensuring consistency e.g., when it comes to date shifts or project-specific patient identifiers (see Section “De-identification”).

#### Deployment and security considerations

Data providers manage large volumes of sensitive patient data, including hospital internal patient identifiers. For security reasons, the SPHN Connector is designed to be installed and operated directly within the local environment of the data provider. The setup and deployment processes are transparent and compliant with the data provider’s security requirements. These requirements are supported by key technical features, including Docker rootless mode; offline and air-gapped execution, ensuring that runtime changes are forbidden; a dedicated Application Programming Interface (API) to automate processes within a workflow; and integrated user and password management for authenticated access.

#### Computational performance and scaling

The SPHN Connector is designed to run efficiently on standard computational infrastructures without requiring specialized hardware or complex system dependencies. It operates in environments with a minimum of 16 GB of RAM, 4 CPU cores, and 500 GB of disk space.

From an operational perspective, to prevent overlapping patient files, the SPHN Connector processes data on a per-patient basis. This ensures consistency and simplifies downstream data handling, for example during delta loads or consent revocation (see Section “Streamlined data updates and deletions using named graphs”).

Core libraries were carefully selected and optimized:

- For parsing and validating RDF data, LightRDF [34] was integrated, a high-performance parser implemented in Rust and accessible via Python bindings.
- For JSON Schema validation, the jsonschema [35] library was chosen for reliability and efficiency.
- For the RDF conversion, RML [36] (RMLmapper-java [37]) was chosen to convert the internal JSON files into RDF files based on the RML configurations. Its flexible RML mapping language extends R2RML [38] to handle multiple data sources (e.g., JSON, CSV, XML), while performing and scaling well.
- For RDF validation, the SPHN RDF Quality Control (QC) framework [39] was used, a Java-based solution adapted within the SPHN Connector to 1) check schema compliance using SHACL constraints and generate a detailed report, and 2) compute quantitative statistics to evaluate data completeness via SPARQL queries.

The SPHN Connector supports integration with external S3 storage for handling large datasets. On the computational side, multi-processor and multi-threaded execution are implemented across all steps, particularly beneficial for computationally intensive tasks such as de-identification and validation. These optimizations support scalability without overloading the local infrastructure while ensuring reliable performance across diverse environments.

### Knowledge graph features

In a federated environment where multiple institutions contribute data, the creation of a reliable KG requires clear design principles. The following subsections describe key decisions implemented in the SPHN Connector that go beyond the semantics specified in the SPHN RDF Schema.

#### IRI naming convention for unique instances

A consistent and collision-free identification of data instances is essential for reliable graph construction. To preserve data integrity, a structured IRI convention is defined [40] and consisting of:

i. The data provider identifier, based on the unique identifier number for enterprises applied in Switzerland (UID [41]).
ii. A prefix specifying the schema from which the instance data originates (e.g., a SPHN or project-specific prefix).
iii. A class name reflecting the type of the instance (defined in the schema).
iv. A unique identifier defined by the data provider for that data instance.

The SPHN Connector automatically fills i, ii and iii when building data instances. This leaves only the last part of the identifier as input to the data provider. This convention guarantees globally unique IRIs, preventing collisions when decentralized data is merged.

#### Linking sample information across providers

Research projects often require linking data elements across institutions (e.g., routine clinical data and cohort data) and domains (e.g., linking electronic health records with omics or imaging data). In SPHN, this is particularly relevant for samples, where a sample collected at a hospital might be analyzed by a research center. Without coordination, identifiers for the same sample remain distinct, preventing linkage across datasets. In SPHN, the support for such linkage is achieved through the property sphn:hasSharedIdentifier, which is exempt from the IRI naming convention. Data providers can agree on a shared identifier and populate it during data ingestion. Patient identifier linkage is also possible; however, researchers are responsible for obtaining the necessary legal and ethical approvals, including clearance from relevant ethics committees and data protection authorities.

#### Streamlined data updates and deletions using named graphs

A key objective of the SPHN Connector is to support evolving patient records by enabling delta loads without reprocessing entire datasets. For instance, when a patient has a new hospital visit or additional lab results become available, only the new information needs to be incorporated. Likewise, if a patient revokes consent, all data associated with that individual must be removed. This is achieved by organizing RDF data into named graphs, with each patient’s data contained within a separate ‘subgraph’, enforcing patient-level atomicity. To support this, the SPHN Connector produces RDF files in TriG and N-Quads formats. In downstream pipelines using the generated KGs, updates are more efficient, targeting individual patient graphs without the need to reload data for all patients. This design also simplifies the handling of consent revocation: a patient’s dataset can be removed by deleting the corresponding named graph via SPARQL.

While design choices improve scalability and data management, ensuring patient data privacy and integrity remains critical. The following section describes the measures implemented in the SPHN Connector to address these aspects.

### Measures for data integrity and privacy

#### De-identification

The SPHN Connector provides integrated de-identification capabilities, including pseudonymization, to support institutions that may lack such functionality in their own data-processing pipelines, particularly cantonal hospitals and smaller data providers. These features enable systematic protection of personally identifiable information. De-identification rules are defined in a project-specific JSON configuration file specified by the data provider. The SPHN Connector currently implements several de-identification functionalities:

- **Field scrambling:** Generates unique, pseudonymized identifiers for selected fields, typically those containing unique identifiers. The resulting values cannot be traced back to their original form but remain consistent within a dataset.
- **Date shift:** All date values associated with concepts (e.g., Birth, Admission, Diagnosis) are shifted by a random number of days selected from a range defined in the configuration. The shift is applied consistently across all records of a patient, which preserves temporal relationships.
- **Field substitution (list or regex):** One or more fields are replaced with a user-defined substitution or placeholder. Sensitive values can be identified either by explicitly listing them or by specifying a regular expression pattern. Any listed or regex-matching value is replaced with a corresponding replacement string.

The SPHN Connector applies de-identification using a Universally Unique Identifier (UUID)-based function [42] with a salting mechanism to support parallelizing UUID generation but does not rely on any patient information. It stores a comprehensive log of data modifications (i.e., a mapping of the patient’s identifier with the de-identified identifier), only accessible to the data provider operating the tool. These logs enable data providers to apply the same de-identification to subsequent data conversions for the same patient. Alternatively, logging can be disabled, which is suitable only for one-off de-identified data preparation and export.

#### Data integrity through validation

Data validation is crucial to guarantee both syntactic correctness and semantic coherence, ensuring consistent modeling and interpretation of heterogeneous data. The SPHN Connector implements multiple validation steps throughout the data processing workflow.

The first layer of validation ensures that incoming data conforms to the expected structure and basic constraints. For JSON data, this is achieved through JSON Schema validation, which checks data types, required fields, and structural rules. For relational tables, Structured Query Language (SQL) constraints enforce similar checks on the table and data type definitions. For example, value sets defined in the RDF schema are mapped to specific data types in PostgreSQL [43], restricting ingestion to permitted values only. This pre-validation catches structural inconsistencies or type mismatches early, reducing downstream errors during data transformation and RDF generation. In addition, a pre-check step ensures that certain fields are correctly formatted (e.g., a value is a valid IRI, without spaces or invalid characters).

While the RDF schema defines the expected data structure, it does not enforce compliance. This is where SHACL plays a crucial role in translating the RDF schema into machine-actionable validation constraints. SHACL constraints define allowed values, cardinalities, and coding expectations, enabling the automated detection of both structural (e.g. missing mandatory metadata) and semantic inconsistencies (e.g. use of outdated or incorrect codes in the data). These SHACL rules are automatically generated during project setup based either on the schema provided as input, using the Python-based SHACLer tool (see [25] for the detailed methodology for SHACL rule creation). It is important to note that the SHACL rules generated primarily ensure data integrity and compliance with the schema restrictions but do not enforce data correctness (e.g., no error is raised when a patient has a systolic blood pressure of 300). The only exception is a logic check which ensures that the start date of an event occurs before its end date.

With validation and de-identification strategies in place, the SPHN Connector orchestrates a structured workflow to transform and deliver semantically compliant and validated RDF data. The following section outlines this workflow from data ingestion to data export.

### Data workflow in SPHN Connector

**Fig. 3:**
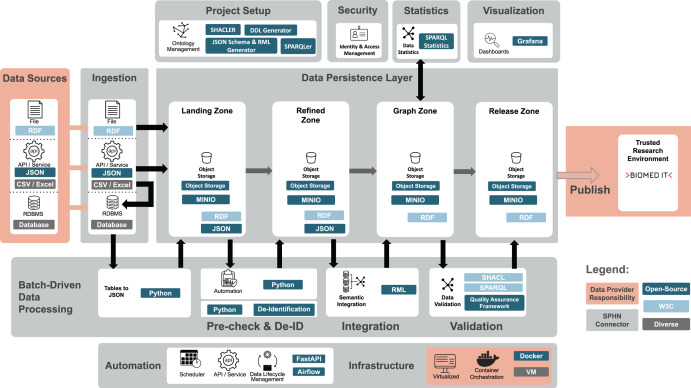
Data architecture and workflow in the SPHN Connector. Patient data from diverse sources (RDF, JSON, CSV/Excel, RDBMS) can be ingested into the SPHN Connector and transformed via RML into SPHN-compliant RDF. Validation through SHACL and SPARQL ensures semantic compliance before datasets are sent to projects via secure infrastructures

The SPHN Connector API, built with FastAPI [44], serves as an entry point for most operations (see complete workflow in Fig. 3) in SPHN. During project setup, users provide the SPHN RDF Schema, optionally complemented with a project-specific RDF Schema extension, together with relevant external terminologies, and specify the desired RDF serialization format (e.g. Turtle, N-Quads, TriG). An optional JSON-based de-identification file may be supplied. At this stage, external terminologies are loaded as static elements to reduce computational overhead during later steps.

Based on the schema, the SPHN Connector automatically generates all components, including the input mapping interfaces (i.e., JSON Schema, CSV/Excel templates, and Data Definition Language (DDL) statements). The design of these templates is documented in the SPHN Connector user guide [45] and enables data providers to prepare their local data marts in alignment with SPHN semantics (i.e., semantic mapping of the clinical data to the requested semantics in the schema specifications), which must be done prior to ingesting data into the tool. The API supports five ingestion interfaces: RDF, JSON, CSV, Excel, and relational database (RDBMS).

**Fig. 4:**
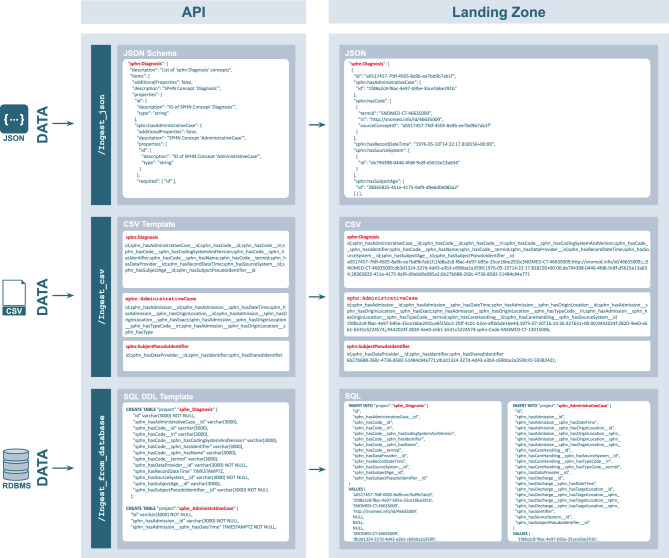
SPHN Connector ingestion interfaces. The figure shows ingestion interfaces for JSON, CSV and Database (three of the five possible interfaces) provided by the SPHN Connector API and how data in the ‘Landing Zone’ could look like once it has been ingested by the API

Central to the schema are “core concepts”, defined as concepts directly linked to the patient (e.g., the Subject Pseudo Identifier). The mapping templates explicitly reflect these core concepts for consistent data modeling. For example, in the SQL DDL (tabular) template, each core concept is represented as a separate table, with one column for each metadata element defined in the schema. When a core concept is linked to another core concept, only the identifier of the linked concept is included in the referencing table, while the full metadata for the linked concept is included in its own dedicated table. For example, ‘Diagnosis’ and ‘Administrative Case’ are two concepts, each directly linked to the ‘Subject Pseudo Identifier’. As such, the SPHN Connector generates separate tables for each concept. The ‘Administrative Case’ concept is associated with the ‘Diagnosis’ concept to capture that a diagnosis was given in a specific hospital administrative case. In this case, the ‘Diagnosis’ table includes a column for the identifier of an ‘Administrative Case’ instance (see Fig. 4). This identifier should correspond to one of the entries in the ‘Administrative Case’ table, which contains all associated metadata for that specific case (e.g., Admission, Discharge). Note that ‘Admission’ is not a core concept, therefore all the metadata associated with the ‘Admission’ is directly embedded in the table of ‘Administrative Case’. These approaches enable the reflection of graph-like relationships in a tabular structure, where links between entities are represented through identifiers rather than nested or interconnected data while optimizing the number of columns populated.

Data can be submitted patient by patient or in batches via (external) S3-compatible or MinIO (within the SPHN Connector) storage. The SPHN Connector also provides a pgAdmin interface for data providers to securely connect, manage, and monitor the underlying PostgreSQL database. The SPHN Connector follows a data lake architecture in which records move through defined zones and are stored as files (see Fig. 3), allowing data inspection at different processing stages. After ingestion, data is normalized into an intermediate JSON format, to support automated quality-control checks when data reaches the ‘Landing Zone’, including validation of code and terminology mappings as well as verification of IRIs. At this stage, de-identification rules in line with project specifications can be applied.

Data is then converted into RDF using RML mappings, to align with the provided schema. The transformed data undergoes SHACL and SPARQL-based validation against the schema and standard terminologies previously loaded. A detailed log is generated allowing users to track errors, warnings, and failing patient records. The generated RDF patient data (even those failing validation) are transferred to the ‘Release Zone’, where users can download RDF data and the associated quality control reports.

Throughout the workflow, processes are monitored and orchestrated using Apache Airflow [46], a workflow execution and management platform, for traceability and supporting systematic debugging.

## Results

### The SPHN Connector

The SPHN Connector implements a schema-driven data transformation pipeline for federating knowledge graphs by producing RDF data locally at each institution. This approach addresses key challenges in cross-institutional biomedical data integration, including semantic interoperability, identifier uniqueness, updatability, and patient-level data governance. In our practical implementation, the tool adheres to the SPHN Semantic Interoperability Framework and its specific RDF schema [22]. Heterogeneous source elements from diverse formats (e.g. CSV, Excel, JSON, relational databases) are mapped to semantically enriched RDF triples/quads. The generated triples/quads undergo validation for semantic correctness, including adherence to SPHN naming conventions and alignment with international terminologies using SHACL constraints. The resulting RDF graphs are constructed using W3C standards for linked data, including RDF, RDFS, and OWL, ensuring interoperability and enabling data integration into a unified federated KG. This approach supports data linkage across different modalities (e.g. clinical, omics) and institutions, enabling advanced querying and applications in biomedical research. Although the SPHN Connector operates as a fully automated system from the user’s perspective, it remains fully documented and transparent, allowing detailed inspection and analysis of its internal processes.

### Performance

In a real-world scenario, we benchmarked the SPHN Connector at the University Hospital Zurich (USZ). The system was run in production with 8 CPU cores, 48 GB RAM, and 500 GB disk space to transform data of 120,203 patients from a local relational database into PostgreSQL. The dataset included 101 SPHN concepts spanning a wide spectrum of complexity from the simplest statements such as the categorial “Administrative Sex” data point of a patient (120,203 instances) to highly granular and structure concepts such as “Drug Prescription” (4 million instances), which captures multi-dimensional information deep into the prescribed substance, administration route, timing etc.

From PostgreSQL to JSON conversion (including de-identification with date shift) through validation and delivery into RDF, the process required 3 days and 19 hours (see Table 1). Without de-identification, the conversion was completed in 2 days and 20 hours. This resulted in the production of 2 billion RDF triples across all patients, with individual patient RDF files (in TriG format) averaging 1 MB. The variation in file size reflects clinical data heterogeneity, from patients with minimal encounter records to those with comprehensive longitudinal data spanning multiple clinical domains. On average, each patient required approximately 2.7 s (with de-identification) or 2.1 s (without) of processing time. Validation is the most time-consuming step (48% without de-identification, 36% with) and its duration is correlated with the number of schema violations detected. De-identification is also a time-consuming step (0.8 s vs. 0.1 s pre-check time for one patient on average with and without de-identification). This step is optional, as data providers may use their own solutions to de-identify data prior to ingestion into the SPHN Connector to accelerate the transformation process.

**Table 1:** Duration of each SPHN Connector phase at USZ in two separate runs

a) Run 1	Duration all patients	Duration all patients (in seconds)	Average duration per patient (in seconds)
PostgreSQL to JSON conversion	**01d 01:43:49**	92 629	0.771
Pre-check & de-identification	**01d 01:48:45**	92 925	0.773
Integration	**00d 06:35:21**	23 721	0.197
Validation	**01d 09:00:16**	118 816	0.988
Total	03d 19:08:11	328 091	2.729
**b) Run 2**	**Duration all patients**	**Duration all patients (in seconds)**	**Average duration per patient (in seconds)**
PostgreSQL to JSON conversion	**01d 01:43:49**	92 629	0.771
Pre-check	**00d 03:13:36**	11 616	0.097
Integration	**00d 06:35:21**	23 721	0.197
Validation	**01d 09:00:16**	118 816	0.988
Total	02d 20:33:02	246 782	2.053

This table reports the execution time of each phase (PostgreSQL to JSON conversion, Pre-check, Integration and Validation), as well as the total processing time for 120,000 patients processed in 12 batches. The average duration of each phase for each patient is also calculated. Two runs were performed: one including de-identification (a) and one without (b). Both runs were executed on an infrastructure with 8 CPU cores, 48 GB RAM and 500 GB of disk space. The processing generated approximately two billion RDF triples, with an average RDF file size per patient of 1 MB (minimum: 12 KB; maximum: 107 MB). Ingestion into PostgreSQL is ignored as this step is heavily dependent on the provider’s setup and tooling

**Fig. 5:**
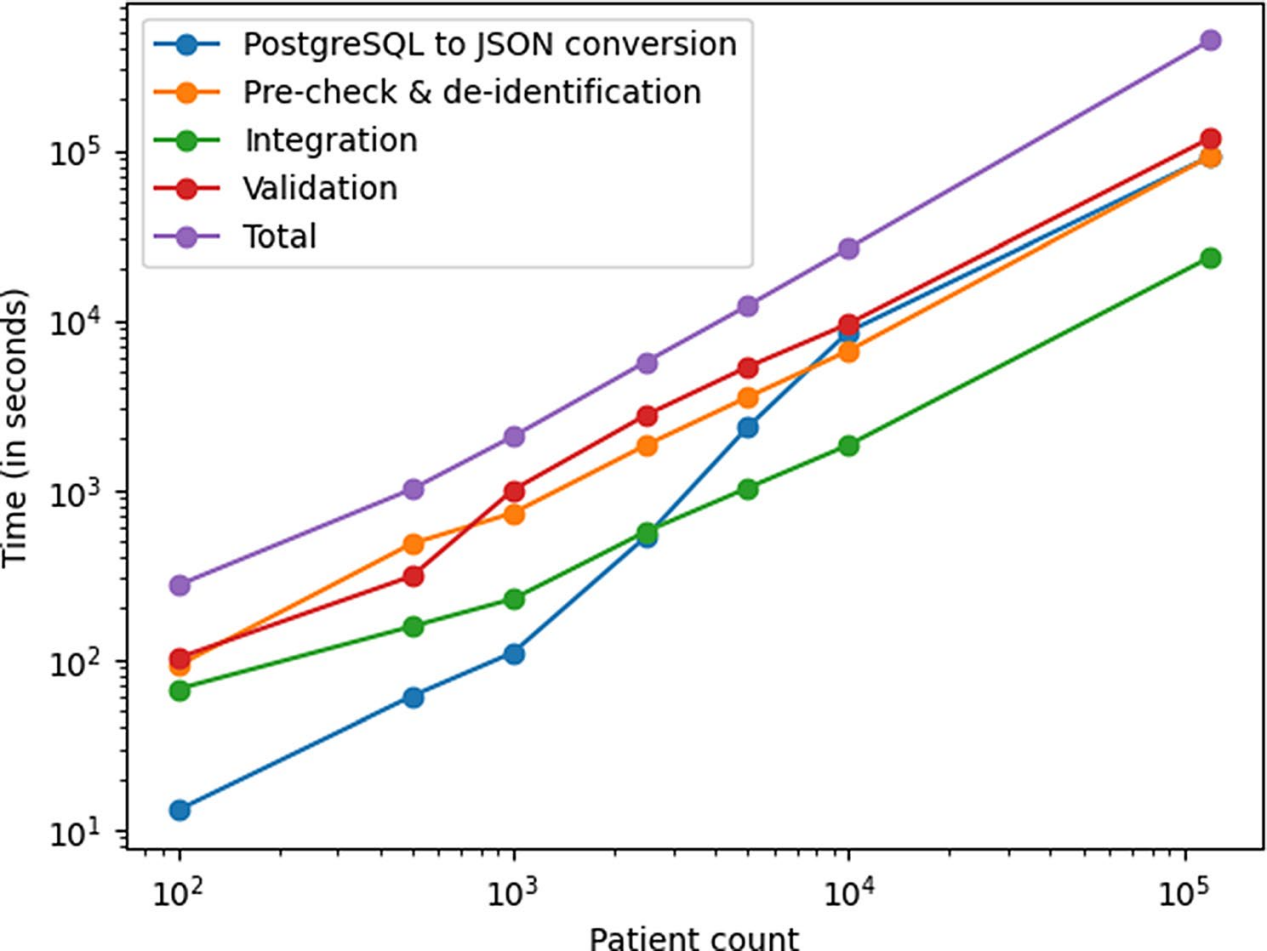
Log-log plot of SPHN Connector execution duration as a function of patient cohort size. The figure shows the runtime (in seconds) of individual phases (PostgreSQL to JSON conversion in blue, Pre-check & de-identification in orange, integration in green, validation in red) and, the total execution time in violet

When benchmarking the SPHN Connector across different patient cohort sizes, the tool scales linearly up to 100,000 patients (see Fig. 5 and Table 2). The average data processing duration per patient stabilizes between 2 and 3 seconds (see Table 2). The results indicate strong scalability with overheads amortized as the cohort size grows. Validation remains the most time-consuming phase across all cohort sizes. However, PostgreSQL to JSON conversion is the least time-consuming phase for up to 1000 patients.

**Table 2:** Duration (in seconds) of each SPHN Connector phase at USZ as a function of patient cohort size

Patient count	100	500	1 000	2 500	5 000	10 000
PostgreSQL to JSON conversion	13	61	110	534	2 343	8 597
Pre-check & de-identification	92	484	732	1 848	3 530	6 632
Integration	67	157	228	571	1 028	1 843
Validation	102	311	1 002	2 780	5 319	9 565
**Total (in seconds)**	**274**	**1 013**	**2 072**	**5 733**	**12 220**	**26 637**
**Average duration per patient (in seconds)**	**2.740**	**2.026**	**2.072**	**2.293**	**2.444**	**2.664**

This table reports the execution time of each processing phase (PostgreSQL to JSON conversion, Pre-check & de-identification, Integration, and Validation), as well as total runtime (sum of the four above rows) and average duration per patient for cohorts ranging from 100 to 10,000 patients (total divided by patient count).

### Deployment and use cases

As of 2025, the SPHN Connector is deployed in production across the clinical data platforms of Swiss university hospitals (University Hospital Basel, University Hospital Bern, Geneva University Hospitals, University Hospital of Lausanne, University Hospital Zurich, and the University Children’s Hospital Zurich) as well as in multiple cantonal hospitals. Through these deployments, the SPHN Connector enabled the creation of multiple project-specific datasets, including the SPHN Federated Clinical Routine Dataset [47], the largest to date, which integrates clinical routine data from more than 800,000 patients across six hospitals into a federated knowledge graph comprising approximately 12 billion RDF triples.

The SPHN Connector is publicly available as open-source software under the GNU General Public License v3.0 (GPLv3), supporting reuse, extensibility, and adoption beyond the SPHN.

## Discussion

The implementation of the above-mentioned strategies reduces operational complexity, minimizes transformation errors, and enhances reproducibility across data deliveries. By embedding validation rules directly into the tool and ensuring that all transformations follow a predictable and version-controlled path, the SPHN Connector fosters institutional trust in data production. Integration with external tools is intentionally modular, enabling the SPHN Connector to operate within diverse workflows while minimizing the impact of changes in external systems. Ultimately, this approach reduces the technical burden on hospital IT teams and improves the quality and interoperability of the data. Once transformed into KGs, data can be used either centrally for integrated data management and analysis or federated for performing cross-institutional analysis while keeping sensitive data local (Fig. 1), depending on governance and operational constraints. Both approaches enable downstream research while respecting local constraints.

Validation emerges as the most time-consuming process in the SPHN Connector, accounting for 48% of the total runtime at USZ. This overhead arises from the systematic data verification against the schema requirements, where millions of data points are checked against the SHACL rules, for consistency and semantic integrity. Unlike other pipeline phases, validation involves complex rule interpretation, including terminology validation against current codes (i.e., for versioned terminologies [48]) and identification of invalid or historically misused codes, and temporal consistency check to ensure a start date precedes or equals an end date. While computationally demanding, this process is crucial for data quality control, especially in federated environments where heterogeneous data sources introduce risks of inconsistencies or errors in downstream analyses. Therefore, validation is a scientific necessity to ensure data trustworthiness and reliability, though it is a key candidate for future optimization to reduce pipeline runtime.

A second performance consideration is the PostgreSQL to JSON conversion, which scales efficiently up to 1000 patients, but shows an increased processing time beyond this threshold. This suggests that the SPHN Connector’s performance is infrastructure-dependent: pipelines with fixed RAM allocations may experience disproportionate slowdowns as patient number increases, highlighting the need for scalable resource provisioning by data providers.

Overall, the execution time, regardless of patient count, remains between two and three seconds per patient in this real-world data benchmarking, which is reasonable for such a robust data transformation pipeline.

While the SPHN Connector addresses a broad range of challenges, it is important to clarify its intended scope and limitations. The tool focuses on data transformation and validation, currently applied to the SPHN use case. It is not a triple store and does not provide capabilities for RDF storage, querying, merging, or reasoning. In addition, the SPHN Connector does not correct errors in source data but validates incoming data against schema definitions and standard terminologies, and reports on inconsistencies. Data providers are responsible for ensuring the accuracy and completeness of the source data. Similarly, the mapping of local terms to standard terminologies requires domain-specific decisions regarding clinical meaning, local coding practices, and institutional semantics. Such decisions cannot be automated centrally without risking misinterpretation or loss of critical information. Hence, terminology mapping must be performed prior to using the tool and lies within the responsibility of data providers. However, emerging approaches based on artificial intelligence and large language models may assist with these tasks and help streamline the process.

During the design and implementation of the SPHN Connector, several discussions were held with stakeholders on possible alternative approaches. These revealed opportunities for improvement as well as important constraints to consider.

Despite the support provided with the SPHN Connector, stakeholders reported that transforming data from clinical data platforms (CDPs) into SPHN-compatible semantics remains a significant challenge, particularly when ingesting tabular data. This difficulty arises both from the integration of heterogeneous data sources into local pipelines and from the complexity of the SPHN RDF Schema, which models healthcare concepts with rich contextual detail to support research use cases. As a result, the tabular DDL templates generated by the SPHN Connector often led to extensive tables with a high number of columns to populate, reflecting the inherently flat structure of tabular formats compared to the interconnected nature of RDF graphs. Nevertheless, most stakeholders acknowledged that the SPHN Connector substantially reduces the burden of adopting Semantic Web technologies by abstracting RDF-specific complexity and providing local validation capabilities. The tool also facilitates migration between schema versions, reducing maintenance effort. By supporting intermediate system-aligned input formats (i.e., tabular, JSON, CSV, Excel), the SPHN Connector simplifies data transformation at the source. Stakeholders further emphasized the value of knowledge graphs for achieving semantic interoperability, particularly when combined with the SPHN RDF Schema, which promotes a shared understanding and consistent data exchange across Swiss institutions.

To ensure long-term maintainability and alignment with evolving semantic standards, we are committed to supporting, with each SPHN Connector release, the latest version of the SPHN RDF Schema, along with project-specific and other actively used schemas within the SPHN ecosystem. Backward compatibility is maintained whenever possible.

One frequently mentioned topic was the horizontal scaling of the tool. While this approach could increase throughput for large datasets, the diversity of local environments poses challenges for deploying and maintaining such solutions. Stakeholders also considered the possibility of connecting the tool directly to the hospital’s CDPs to avoid intermediate data transfers and streamline workflows. However, this would require highly customized versions of the SPHN Connector for each CDP. Hospitals also expressed concerns about the security implications of granting direct access to CDPs, as this could bypass established governance. Consequently, a more decoupled approach was considered safer and broadly acceptable.

Finally, we considered processing all data collectively, without partitioning it into patient-specific workflows. While this approach could offer performance advantages, it increases the risk of generating overlapping or inconsistent patient files and complicates debugging. The decision to adopt a patient-oriented workflow was driven by the need for operational simplicity and consistent data handling across participating sites.

Overall, the implemented decisions reflect a balance between technical robustness and operational feasibility, fostering stakeholder trust while enabling a reliable and transparent RDF data transformation pipeline.

## Conclusion

The SPHN Connector exemplifies how, from an architecture- and use case-driven approach, we implemented a tool that facilitates the construction of KGs from heterogeneous health data systems across Swiss healthcare institutions. Its development goes beyond simple data transformation, integrating KG-focused design principles to ensure semantic consistency, identifier uniqueness, and interoperability across sites. By operationalizing the shared SPHN RDF Schema and a consistent, globally unique IRI naming and identifier management strategy, the SPHN Connector provides the syntactic and semantic guarantees needed for conceptual unification. Looking forward, the federated integration of knowledge graphs across domains and institutions represents a promising direction to not only enable broader data reuse but also address complex governance and data-sharing challenges.

## Data Availability

Tool name: SPHN Connector. Tool home page:https://git.dcc.sib.swiss/sphn-semantic-framework/sphn-connector. Operating system(s): Linux, Windows. Main programming language: Python. Other requirements: Docker. License: GNU GPL v3.0.

## Abbreviations

API: Application Programming Interface
ATC: Anatomical Therapeutic Chemical
CDP: Clinical Data Platforms
CHOP: Swiss Classification of Surgical Interventions
CPUs: Central processing units
CSV: Comma Separated Values
DDL: Data Definition Language
EHDEN: European Health Data & Evidence Network
ETL: Extract Transform Load
FAIR: Findable Accessible Interoperable Reusable
FHIR: Fast Healthcare Interoperability Resources
GB: Gigabyte
KB: Kilobyte
KG(s): Knowledge Graph(s)
ICD-10-GM: International Statistical Classification of Diseases and Related Health Problems 10th revision German modification
IRI: Internationalized Resource Identifier
JSON: JavaScript Object Notation
MB: Megabyte
OBO: Open Biological and Biomedical Ontology
OHDSI: Observational Health Data Sciences and Informatics
OMOP: Observational Medical Outcomes Partnership
RAM: Random Access Memory
RDF: Resource Description Framework
SERI: Swiss State Secretary of Research and Innovation
SHACL: Shapes Constraint Language
SQL: Structured Query Language
SPARQL: SPARQL Protocol and RDF Query Language
SPHN: Swiss Personalized Health Network
UID: Unternehmens-Identifikationsnummer (in english: standardised business identification number)
URI: Uniform Resource Identifier
USZ: University Hospital Zurich
